# Impact of pumpkin seeds supplementation on anemia in antenatal mothers at Andhra Pradesh, India

**DOI:** 10.6026/973206300181092

**Published:** 2022-11-30

**Authors:** Swapna Kumari Andugula, Veerraju Andugula, Mani Vadivel, Nithya Rajapandian, Chaitra Jinka, Shyamaladevi Babu, A Gouri Shankar

**Affiliations:** 1 Saveetha College of Nursing, Saveetha Institute of Medical and Technical Sciences (SIMATS), Chennai, Tamilnadu, India; 2 KIMS College of Nursing, KIMS and RF, Amalapuram, East Godavari district, Andhra Pradesh, India; 3 APR School (boys), Maredumilli, Rajahmundry, East Godavari district, Amalapuram, Andhra Pradesh,India; 4 Department of Biochemistry, Arunai Medical College and Hospital, Tiruvanamallai- 606603, Tamilnadu, India; 5 Department of Physiology, Arunai Medical College and Hospital, Tiruvanamallai- 606603, Tamilnadu; 6 Syngene International Limited, Biocon park, Bangalore, Karnataka, India; 7 Faculty of Allied Health Sciences, Chettinad Hospital and Research Institute, Chettinad Academy of Research and Education, Kelambakkam-603103, Tamil Nadu, India; 8 General Medicine, Saveetha Medical College, Saveetha Institute of Medical and Technical Sciences (SIMATS), Chennai, Tamilnadu, India

**Keywords:** Antenatal mothers, anemia, nutritional status, pumpkin seeds, demographic, obstetric variables

## Abstract

Anemia during pregnancy causes 40% of maternal fatalities in underdeveloped nations, according to statistics from the WHO. Pregnant women should meet the requirements for various nutrients, notably micronutrients, to ensure a better pregnancy outcome in the future. Cucurbita moschata (Pumpkin) seeds contain a variety of compounds, such as m-carboxyphenylalanine, amino butyrate, and citrulline, as well as a number of other amino acids. It has a wide variety of biological activity such as antioxidant, anticancer, anti-inflammatory etc. However, the community's use of pumpkin seeds is still limited. The present study was aimed to assess the impact of supplementation of pumpkin seeds on anemia among antenatal mothers in relation to Demographic and obstetric variables at Amalapuram Mandal, East Godavari district, Andhra Pradesh. In the third stage of the study, antenatal mothers' pre- and post-test scores for indicators of anaemia, pica, nail health, level of activity, heart rate, capillary refill, nutritional status, extremities, and level of hunger were compared. According to age, education, occupation, monthly income, and information source, the data demonstrates that according to three prenatal moms' consumption of pumpkin seeds in the morning, three in the afternoon, and one in the evening. The data available indicates that, of the 5 prenatal moms, 2 displayed symptoms of anemia with respect to the length of the menstrual cycle, the volume of menstrual flow, and the method of the previous delivery. Additionally, it was discovered that three of the pregnant mothers had appropriate hemoglobin levels and three of them had inadequate levels based on their past deliveries, menstrual flow volume, and cycle length. It also revealed that there is no discernible relationship between pregnant mothers in Amalapuram Mandal's diet of dry fruits and nuts and menstrual cycle length, flow volume, or method of prior birth.

## Background:

Pregnancy is the period from conception until the start of labour during which the foetus develops and grows inside the mother's womb. The average gestational period is thought to be 280 days (40 weeks), with a maximum of 300 days (43 weeks). Anemia during pregnancy causes 40% of maternal fatalities in underdeveloped nations, according to statistics from the WHO [1]. The main causes of anemia in pregnancy are iron deficiency, severe bleeding, and occasionally even their interaction. One of the many elements that affect iron metabolism is protein) [2]. Protein is a major factor in assisting with organic iron intake. For binding iron and preventing potentially hazardous oxidants in the form of hemoproteins as heme molecules (haemoglobin [Hb] or myoglobin) and heme enzymes, organic iron is a crucial functional component. Organic iron can be found in food, especially in red meat. Serum contains ferritin, a protein that stores iron extra cellularly. Ferritin is a medical indicator of the condition of the body's iron reserves. Iron deficiency anaemia dramatically increased platelet count (PT) and overall iron-binding capacity (TIBC) [3-4]. Pregnant women should meet the requirements for various nutrients, notably micronutrients, to ensure a better pregnancy outcome in the future. Pregnant mothers and their unborn children can meet their nutritional needs by consuming the nutritious pumpkin fruit. Pumpkin seeds are still only used in the community [5]. Cucurbita moschata seeds contain a variety of compounds, such as m-carboxyphenylalanine, amino butyrate, and citrulline, as well as a number of other amino acids needed by the prostate gland, such as seminal alanine, glycine, and glutamic acid. These seeds also include the minerals Zn (zinc) and Mg (magnesium), which are essential for reproductive health. Pumpkin seed phytoestrogens may have estrogenic or anti-estrogenic properties that reduce blood pressure and C-reactive protein (CRP) [6]. It contains a variety of biological properties, including antioxidant, anti-inflammatory, and anti-cancer properties [7]. Thus, in the current study, the influence of pumpkin seed supplementation on anaemia among pregnant mothers in Amalapuram Mandal, East Godavari district, Andhra Pradesh, was examined.

## Methodology:

## Research approach and design:

Experimental research approach was chosen as the study's research strategy. The experimental research design was chosen for the current investigation.

## Description of variables:

The variables of the study are as follows:

## Independent variable:

Effects of adding pumpkin seeds to pregnant women's diets on anaemia in such women

## Dependant variable:

Level of anemia among antenatal mothers

## Demographic variables:

The source of information on the effects of prenatal food supplementation of pumpkin seeds on anaemia among pregnant moms, as well as demographic factors including age in years, religion, education, occupation, family income per month in rupees, type of family, and age in years.

## Obstetric variables:

The effects of prenatal food supplementation with pumpkin seeds on anaemia among antenatal mothers were studied in relation to the age of menarche, frequency of menstrual cycles, duration of menstrual cycles, amount of menstrual flow, gestational age in weeks, date of antenatal visit, gravida, para, and mode of previous delivery.

## Setting, population and sample size:

East Godavari district in Andhra Pradesh's rural Amalapuram Mandal serves as the study's location. There are 83214 people living in Rural Amalapuram Mandal. 14 prenatal mothers from Rural Amalapuram in Andhra Pradesh's East Godavari district made up the study sample.

## Sampling and method of data collection

The data are gathered with a straightforward random sampling technique. A systematic questionnaire is used to collect the data for the current investigation. It includes the pre-designed responses and the predefined set of questions that were typically answered in a predetermined order. The questionnaire will be employed since it is the best practical data collection tool for the research project to get the needed factual data.

## Development and description of tools:

In order to design an appropriate tool for evaluating knowledge on the effects of prenatal food supplementation of pumpkin seeds on anaemia among pregnant moms, a literature search was conducted. With the use of selected material from a variety of text books, journals, the internet, and discussions with professionals in the fields of nursing, obstetrics, and community medicine, an instrument in the form of a structured questionnaire will be constructed.

## Criteria for sample selection:

The selection criteria for the sample are mostly represented as inclusion and exclusion criteria.

## Inclusion Criteria:

The prenatal mothers ranged in age from 19 to 34.

 Pregnant women whose gestational ages range from 16 to 36 weeks.

 Mothers who had one, two, three, or more children were all included.

The grand multipara, multiple, and primi mothers were all included.

Antenatal mothers who are literate in Telugu and English and can read and write it.

## Exclusion criteria:

 Pregnant women with high-risk pregnancies; • Pregnant women who are unwell.

 People who don't cooperate

People who don't live in Amalapuram Mandal

## Validity of tool:

The tool was presented to community and obstetrics professionals in order to assess the content validity. Modifications will be made as appropriate after receiving the insightful ideas.

## Reliability of tool:

The test-retest approach will be used to evaluate the tool's dependability.

## Pilot study:

Development and description of tool:

A study was conducted to learn more about the effects of antenatal mothers' consumption of pumpkin seeds as a prenatal food supplement on anaemia. The structured questionnaire was created using conversations with experts in the fields of nursing and obstetrics & gynaecology as well as selected material from a variety of text books and magazines.

## Methods of data collection:

## xPart A:

It discusses the demographic factors that affect mothers' knowledge, including age, religion, education, occupation, family income per month, family structure and the source of information on the benefits of prenatal dietary supplementation with pumpkin seeds on anaemia in expectant mothers.

## Part B:

It discusses the effects of prenatal food supplementation with pumpkin seeds on anaemia in expectant mothers as well as the age of menarche, frequency of menstrual cycles, duration of menstrual cycles, amount of menstrual flow, gestational age in weeks, date of antenatal visit, gravida, parity, and mode of previous delivery.

## Part C:

It focuses on a well-structured questionnaire on anaemia knowledge.

## Plan for data analysis:

## Descriptive statistics:

The mean, standard deviation, frequency distribution, and percentage distribution are the descriptive statistics employed in the study.

## Inferential statistics:

Sign Wilcoxon rank test, t-test, and chi square test are the inferential statistics applied to the investigation.

## Ethical Consideration:

Before beginning the study, written approval from the Amalapuram local government was acquired, as well as the subjects' informed consent.

##  Results and Discussion:

The prenatal moms from Rural Amalapuram in Andhra Pradesh's East Godavari district make up the study sample. The data are gathered using a random sampling method. A systematic questionnaire is used to collect the data for the current investigation. It includes the pre-designed responses and the predefined set of questions that were typically answered in a predetermined order. The questionnaire will be employed because it is the most suitable and practical method for acquiring the needed factual data for the research project [8]. The overall profile of the 14 people who were chosen for the pilot study was analyzed, and the results are shown in Table 1(see PDF). The age distribution of the subjects showed that the majority, or 65%, are between the ages of 23 and 26, 14% are between the ages of 19 and 22, 27 and 30, and 7% are between the ages of 31 and 34. It demonstrates that adult women made up the majority of the sample. According to the data, the majority of participants (64% of them) identify as Hindus, 36% as Christians, and none as Muslims or anyone else. In terms of educational status, the majorities (36%) of the subjects are in secondary and intermediate education, 21% are graduates, and 7% are illiterate, which may be because of poverty and a psychology of hard-earned subsistence that prevents them from understanding the importance of female education. It also demonstrated that parents have little to no interest in their daughters' educational pursuits. In sample areas, there are a number of socioeconomic reasons that are both directly and indirectly to blame for the high dropout rate and poor literacy rate among pregnant women. It also showed that the parents genuinely show no concern for their daughters' educational needs. The high dropout rate and poor literacy rate among pregnant women in sample areas are caused by a number of socioeconomic factors, both directly and indirectly.

 The National Statistics Institute's (INE) socioeconomic status, which relates yearly household income and average income per consumption unit, can be connected with the socioeconomic status variable, which includes income, education, and employment [9, 10]. As a result, the socioeconomic position of the research participants' women may be divided into four groups: Low (Rs. 5000/-), Medium (Rs. 6000/- to Rs. 10000/-), Medium-high (Rs. 11000/- to Rs. 15000/-), and High (> Rs. 16000/-). According to Table 1(see PDF), the majority of families earn between Rs. 11,000 and Rs. 15000 per month, followed by Rs. 6,000 to Rs. 10,000 and Rs. 5,000 to Rs. 5,000 per month. No family earns more than Rs. 16,000 per month. In terms of occupation, 50% of those surveyed are housewives, 21% work for the government or for businesses, 8% are employed privately, and none are self-employed.

The quality of the marital and family environments is particularly important for the aetiology of prenatal mental disorder. Family structures are significant understudied potential moderators; hence they are mentioned in the current study as well. According to the information about family type in Table 1(see PDF), the nuclear family system is replacing the joint family system due to the speed of time and urbanisation. 56.6% of the population is from a nuclear family, 29% is from a joint family, and 15% is from an extended family. The majority of anaemic adolescent girls (56.7%) were raised in nuclear households, while the remainder anaemic adolescent girls resided in joint houses, according to Sachan et al. (2012). When looking at the sources of information, the majority (72%) is from health care providers, the media and family members (14%), none are related to the friends and others.

The information in the Table 2(see PDF) above showed what the expectant mothers experienced. Regarding menarche age, the majority, or 79%, belong to 13 or under, while 21% belong to 13 or over. 71% of women have menstrual cycles that last longer than 28 days, whereas 29% have cycles that last less than 28 days. The majority of women, or 43%, have menstrual cycles that last between 4 and 5 days, 14% have cycles that last less than 3 days, and none have cycles that last longer than 5 days. The data also explains the menstrual flow, showing that 65% of women use 6 pads (40 ml), 21% use 4 pads (20 ml), 14% use 8 pads (60 ml), and none use 2 pads (10 ml). In terms of gestational age in weeks, 44% fall between 16 and 25 weeks, 28% between 26 and 30 weeks, and 31% between 31 and 36 weeks. The data also includes information about prenatal visits, of which 43% are between 12 and 25-36 weeks and 14% are between 13 and 24 weeks. Sixty-four percent of women are gravida, 36 percent are gravida two, and none are gravida three, four, or more. Regarding parity, the majority, or 57%, belong to the 1 to 3 parity range, the 43% belong to the >5 parity range, and none belong to the 4 to 5 parity range. The bulk, or 64%, of past deliveries were made via caesarean section, whereas 36% occurred naturally through the vaginal canal. In the third stage of the study, pregnant mothers in the Amalapuram mandal were evaluated for indicators of anaemia, pica, nail health, level of activity, heart rate, capillary refill,nutritional status, extremities, and level of appetite, represented in Table 3-12(see PDF). According to the findings, 43% of moms had no symptoms of anaemia before the test, while 57% of mothers did. After the test, 100% of the subjects showed no evidence of anaemia, and none of them did. Regarding pica, 29% of moms had symptoms during the pre-test, whereas 71% of mothers had none. In the post-test, each and every one of them had no evidence of pica. In this study, the health of nails is also taken into account, and the results demonstrate that in the pre-test, all moms had healthy nails and that none of them had spoon-shaped or fragile nails. The pre-test study revealed that 29% of mothers experienced fatigue and general weakness, while 71% of moms engaged in usual levels of activity. 100% of people participated in the post-test at a regular level.

Regarding heart rate, in the pre-test period, 58% of mothers had normal heart rates, 42% had irregular heart rates, and 100% had normal heart rates in the post-test period. Insufficient capillary refill affected 100% of moms; none of them had it in the pre-test, but 100% of them had it in the post-test. Pre-test nutritional status showed that 43% of mothers had appropriate nutrition, while 57% had poor nutrition, and post-test nutritional status showed that 100% of mothers had adequate nutrition. 100% of moms had no numbness or tingling in their extremities prior to the test, and none of them experienced it following the test. In the pre-test, all moms had a healthy appetite, and none had a weak appetite.

In this study, the food pattern was also examined. In the pre-test period, 71% of moms consumed green leafy vegetables, while 29% did not, whereas in the post-test period, 100% of mothers consumed green leafy vegetables. In terms of meat eating, 57% of mothers consumed meat and 43% did not during the pre-test. In the post-test, 86% of mothers reported eating meat, while 14% reported eating no meat. In the pre-test period, 43% of mothers consumed liver, while 57% did not; in the post-test period, 43% of mothers consumed liver, while 57% did not. When it comes to the consumption of citrus fruits, 71% of moms did so prior to the test, while 29% did not, while 100% of mothers did so following the test. Taking Tea/Coffee Consumption into Account 43% of mothers didn't drink any tea or coffee before the test, whereas 57% did. In the post-test period, 43% of moms reported drinking tea or coffee, whereas 57% reported not doing so. The aforementioned data also reveals that, in the pre-test period, 71% of mothers consumed dry fruits and nuts, while 29% did not, and that, in the post-test period, 100% of mothers consumed dry fruits and nuts.

Regarding the consumption of eggs and beans, 71% and 57% of moms consumed eggs and beans, while 29% did not consume either during the pre-test. In the post-test, 86% and 71% of moms reported consuming eggs and beans, while 14% and 29% reported not consuming either. 86% of moms consumed sprouts throughout the pre-test week, while 14% did not. In the post-test, all mothers had sprout consumption. According to the aforementioned data, 43% of mothers consumed pumpkin seeds in the morning, 43% in the afternoon, and 14% in the evening. According to age, education, occupation, monthly income, and information source, the data demonstrates that according to three prenatal moms' consumption of pumpkin seeds in the morning, three in the afternoon, and one in the evening.

The data available indicates that, of the 5 prenatal moms, 2 displayed symptoms of anaemia with respect to the length of the menstrual cycle, the volume of menstrual flow, and the method of the previous delivery. Additionally, it was discovered that three of the pregnant mothers had appropriate haemoglobin levels and three of them had inadequate levels based on their past deliveries, menstrual flow volume, and cycle length. In Amalapuram Mandal, 5 antenatal mothers consumed green leafy vegetables, 2 did not, when taking into account the length of the menstrual cycle and the method of the previous delivery. In contrast, when considering the volume of the menstrual flow, 4 antenatal mothers consumed green leafy vegetables, 3 did not. Consumption of green leafy vegetables had no discernible relationship to menstrual cycle length, menstrual flow volume, or method of prior delivery. When taking into account the length of the menstrual cycle, the volume of menstrual flow, and the method of the previous birth, 4 prenatal moms consumed dry fruits and nuts, whereas 3 did not. There is no discernible relationship between pregnant mothers in Amalapuram Mandal's diet of dry fruits and nuts and menstrual cycle length, flow volume, or method of prior birth.

## Conclusion:

The present study revealed that, there is no discernible relationship between consumption of dry fruits and nuts and menstrual cycle length, flow volume, or method of prior delivery.
